# Burden of communicable and non-communicable diseases-related inequalities among older adults in India: a study based on LASI survey

**DOI:** 10.1186/s12877-022-03481-x

**Published:** 2022-10-10

**Authors:** Shekhar Chauhan, Shubham Kumar, Ratna Patel, David Jean Simon, Aradhana Kumari

**Affiliations:** 1 Department of Family and Generations, International Institute for Population Sciences, Mumbai, India; 2 Department of Survey Research and Data Analytics, International Institute for Population Sciences, Mumbai, India; 3 Research Scholar, Department of Public Health and Mortality Studies, International Institute for Population Sciences, Mumbai, India; 4 Research Scholar, Paris 1 Pantheon Sorbonne University, Paris, France; 5 Research Scholar, Jawaharlal Nehru University, New Delhi, India

**Keywords:** Communicable diseases, Non-communicable diseases, Older population, Double burden, India

## Abstract

**Background:**

A rising proportion of elderly in India has infused notable challenges to the healthcare system, which is already underdeveloped. On one side, NCDs are increasing among the elderly in India; however, on the other side, CDs are also a cause of concern among the elderly in India. While controlling the outbreak of communicable diseases (CDs) remained a priority, non-communicable diseases (NCDs) are placing an unavoidable burden on the health and social security system. India, a developing nation in South Asia, has seen an unprecedented economic growth in the past few years; however, it struggled to fight the burden of communicable and non-communicable diseases. Therefore, this study aimed at examining the burden of CDs and NCDs among elderly in India.

**Methods:**

Data from Longitudinal Ageing Study in India (LASI Wave-I, 2017–18) were drawn to conduct this study. The LASI is a large-scale nationwide scientific study of the health, economics, and social determinants and implications of India's aged population. The LASI is a nationally representative survey of 72,250 aged 45 and over from all Indian states and union territories. Response variables were the occurrence of CDs and NCDs. The bi-variate and binary logistic regression were used to predict the association between communicable and non-communicable diseases by various socio-demographic and health parameters. Furthermore, to understand the inequalities of communicable and non-communicable diseases in urban and rural areas, the Fairlie decomposition technique was used to predict the contribution toward rural–urban inequalities in CDs and NCDs.

**Results:**

Prevalence of communicable diseases was higher among uneducated elderly than those with higher education (31.9% vs. 17.3%); however, the prevalence of non-communicable diseases was higher among those with higher education (67.4% vs. 47.1%) than uneducated elderly. The odds of NCDs were higher among female elderly (OR = 1.13; C.I. = 1–1.27) than their male counterparts. Similarly, the odds of CDs were lower among urban elderly (OR = 0.70; C.I. = 0.62–0.81) than rural elderly, and odds of NCDs were higher among urban elderly (OR = 1.85; C.I. = 1.62–2.10) than their rural counterparts. Results found that education (50%) contributes nearly half of the rural–urban inequality in the prevalence of CDs among the elderly. Education status and current working status were the two significant predictors of widening rural–urban inequality in the prevalence of NCDs among the elderly.

**Conclusion:**

The burden of both CD and NCD among the elderly population requires immediate intervention. The needs of men and women and urban and rural elderly must be addressed through appropriate efforts. In a developing country like India, preventive measures, rather than curative measures of communicable diseases, will be cost-effective and helpful. Further, focusing on educational interventions among older adults might bring some required changes.

**Supplementary Information:**

The online version contains supplementary material available at 10.1186/s12877-022-03481-x.

## Background

South-Asian countries are still young in terms of their demographic profile, but their population is aging as well [1]. South Asian countries face a growing number of healthcare challenges due to their aging populations [1]. In spite of the fact that communicable diseases remain a top priority, non-communicable diseases continue to pose a considerable burden to health care systems [1]. India, a developing nation in South Asia, has seen an unprecedented economic growth in the past few years; however, it struggled to fight the burden of communicable and non-communicable diseases [1]. India, with more than 8 percent of its population being more than 60 + years [2–4], has acquired the level of an ageing nation with the growth of older Indian population in absolute number comparatively faster than other regions of the world [5]. A rising proportion of elderly in India has infused notable challenges to the healthcare system, which is already undeveloped [6]. With a slighter decline in Communicable Diseases (CD) and a steeper increase in Non-communicable disease (NCD), India is currently undergoing the double burden of CDs and NCDs [7]. A critical gap in the health system and policy development was addressed in October 2015 when the India State-Level Disease Burden Initiative was formally launched.

As India undergoes a rapid demographic and epidemiological transition, these processes run parallel [8]. The growing number of NCDs in developing countries such as India has added to the burden of communicable diseases [9]. Currently, India is undergoing double burden of disease, with CDs being a concern at one side, and NCDs being rising on the other side [10]. NCDs are degenerative diseases and are linked to older adults. India is experiencing an increase in the proportion of older people, so NCDs will continue to be a challenge.

CDs were the prime cause of death worldwide for a very long time [11]. NCDs were initially considered diseases of the rich and burdened healthcare systems only in developed countries [11]. Off late, NCDs seemed to be sweeping the globe, a trend that is becoming more prominent in developing countries [11]. India is also experiencing an increasing burden of NCD among elderly [12, 13]. India has been undergoing an epidemiological transition resulting from rise in non-communicable diseases [14]. Despite an increasing burden of NCD, India still does not have sufficiently detailed data on NCDs for research and policy purposes [12]. India's elderly population is on the rise with non-communicable diseases; communicable diseases, however, also pose a threat [7]. The projected increase in the elderly population in India [15] would bring several repercussions as far as the burden of CDs, and NCDs is concerned [7]. In light of the limited literature on CD and NCD among elderly in Indian context, it is imperative to examine the burden of CDs and NCDs in the country. Therefore, this study aimed at examining the burden of CDs and NCDs in a single study among elderly in India. This study explored prevalence and determinants of CDs and NCDs among elderly in India along with examining the urban–rural inequalities in the prevalence of CDs and NCDs among elderly in India.

## Methods

### Data

The study utilizes de-identified data from Longitudinal ageing study of India (LASI), first wave: 2017–18, conducted by the collaboration of International Institute for Population Sciences (IIPS), Harvard T.H. Chan School of Public Health (HSPH), and University of Southern California (USC), and several other national and international institutions. [16]. The survey has been funded by the Ministry of Health and Family Welfare (MoHFW), the Government of India, the National Institute on Aging (NIA), and the United Nations Population Fund, India (UNFPA). The survey included the older adults (men and women) age 45 years and above across all the states (exclude Sikkim) and union territories in India. The LASI wave-1 covers comprehensive aspects of chronic health conditions, functional and mental health, healthcare utilization, family and social networks, work and employment and life expectations.

The LASI has utilized a multistage stratified area probability sampling design to reach out a representative sample. Moreover, rural areas are sampled in three stages while urban areas are sampled in four stages. Further, the first stage engaged to selection of primary sampling units (PSUs) i.e. Tehsils and Talukas. The second stage considered the selection of villages in rural areas and wards in urban areas. In the third stage, household were selected from pre-selected villages in rural areas where census enumeration blocks (CEBs) were selected in urban areas. In additional and final stage of sampling in urban areas, the process of selection of household was made through selected CEBs.

The LASI featured with 72, 250 individuals, including 31,434 aged 60 years and above and 6,749 individuals aged 75 years and above. However, this study considered 60 years and above population for the analytical purpose.

### The double burden of communicable and non-communicable diseases

Double burden of disease refers to the situation where an individual suffers from both non-communicable and infectious diseases. A study classified the burden of diseases in three broad clusters: communicable diseases, non-communicable diseases, and injuries [7]. This study examines the responses of communicable and non-communicable diseases only. Following diseases were included as communicable disease: Jaundice/ Hepatitis, Tuberculosis (TB), Malaria, Diarrhoea/gastroenteritis, Typhoid, Urinary Tract Infection, Chikungunya and Dengue. Within non-communicable diseases, following conditions were included: Hypertension or high blood pressure, diabetes or high blood sugar, Cancer or a malignant tumour, Chronic lung diseases such as asthma, chronic obstructive pulmonary disease/Chronic bronchitis or other chronic lung problems, Chronic heart diseases such as Coronary heart disease (heart attack or Myocardial Infarction), congestive heart failure, or other chronic heart problems, Stroke, Arthritis or rheumatism, Osteoporosis or other bone/joint diseases, Any neurological, or psychiatric problems such as depression, Alzheimer’s/Dementia, unipolar/bipolar disorders, convulsions, Parkinson’s, etc. and High cholesterol.

### Study variables

#### Response variable

The response variables for this study are communicable diseases and non-communicable diseases. Communicable diseases are diagnosed by health professionals and asked as “In the past 2 years, have you had any of the following diseases?” and responses have been recorded in ‘yes’ and ‘no.’ Similarly, non-communicable diseases are also diagnosed by health professionals and asked in the form of ‘yes’ and ‘no.’

#### Predictors

The predictors for this study are considered as sex (male and female); age (60–69 and 70 years and above); marital status (currently married, never married, Divorced/Separated/Deserted/Widowhood), education (No education, below primary, primary, secondary, and higher); living arrangements (living alone, with spouse and with others); place of residence (rural and urban); currently working (yes and no); wealth index (poorest, poorer, middle, richer and richest); self-rated health (poor and good; physical activity (yes and no); tobacco use (no and yes); alcohol use (yes and no); ADL disability (severe, moderate and no disability), and IADL disability (severe, moderate and no disability) [17]. Furthermore, ADL and IADL disability constructed from five (bathing, dressing, mobility, feeding, and toileting) and seven (preparing a hot meal (cooking and serving), shopping for groceries, making telephone calls, taking medications, doing work around the house or garden, managing money, such as paying bills and keeping track of expenses and getting around or finding an address in an unfamiliar place) activities. Both the ADL and IADL disability was categorized into the three categories as “severe,” “moderate,” and “no disability” based on the scale given in previous studies [18, 19]. Further, tobacco use can be defined “ever smoked tobacco (cigarette, bidi, cigar, hookah, cheroot) or used smokeless tobacco (such as chewing tobacco, gutka, pan, masala etc.)”. In addition, alcohol use can be defined as “ever consumed any alcoholic beverages such as beer wine, liquor, country liquor etc.” [13].

### Statistical measures

The analyses were carried out with statistical software STATA version 16. Bivariate technique was used to understand the prevalence of communicable diseases and non-communicable diseases by socio-demographic and health parameters and across various states in India. Further, binary logistic regression was used to predict the association between communicable and non-communicable diseases and socio-demographic and health parameters. We have used enter method of regression where all the predictor variables have been selected in a single step. The equation for binary logistic regression is given below,

$$\mathrm{log}\left(\frac{{p}_{i}}{1-{p}_{i}}\right)=logit\left({p}_{i}\right)={\beta }_{0}+{\beta }_{1}{x}_{1}+{\beta }_{2}{x}_{2}+\dots +{\beta }_{n}{x}_{n}$$

In the above regression equation, ${p}_{i}$ is the probability of being perceived as communicable or non-communicable diseases, ${x}_{1}$, ${x}_{2}$…${x}_{2}$ are the predictors, ${\beta }_{0}$ is the intercept and ${\beta }_{1}$, ${\beta }_{2}$…${\beta }_{n}$ are the coefficients.

Furthermore, to understand the inequalities of communicable and non-communicable diseases in urban and rural areas, the Fairlie decomposition technique was used to predict the contribution toward rural–urban inequalities in CDs and NCDs [20]. The Fairlie technique was first initiated by Fairlie in 1999 which used to estimate from a logit or probit model. The equation for Fairlie decomposition can be written as,

$${\overline{Y} }^{U}-{\overline{Y} }^{R}=\left[\sum_{i=1}^{{N}^{U}}\frac{F({X}_{i}^{U}{\widehat{\beta }}^{U})}{{N}^{U}}-\sum_{i=1}^{{N}^{R}}\frac{F({X}_{i}^{R}{\widehat{\beta }}^{R}}{{N}^{R}}\right]+\left[\sum_{i=1}^{{N}^{R}}\frac{F({X}_{i}^{R}{\widehat{\beta }}^{U})}{{N}^{R}}-\sum_{i=1}^{{N}^{R}}\frac{F({X}_{i}^{R}{\widehat{\beta }}^{R}}{{N}^{R}}\right]$$

where N^U^ and N^R^ is the sample size for urban and rural respectively, ${\overline{Y} }^{U}$ and ${\overline{Y} }^{R}$ are the average probability of a binary outcome of interest for group urban and rural, F is the cumulative distribution function from the logistic distribution, distribution, ${X}_{i}^{R}$ and ${X}_{i}^{U}$ are the set of the average value of the independent variable and ${\widehat{\beta }}^{U}$ and ${\widehat{\beta }}^{R}$ are the coefficient estimates for the urban and rural, respectively. The results can be interpreted as: positive values indicate rural–urban inequalities in CDs or NCDs where negative values show the lower inequality for the same.

## Results

**Fig. 1:**
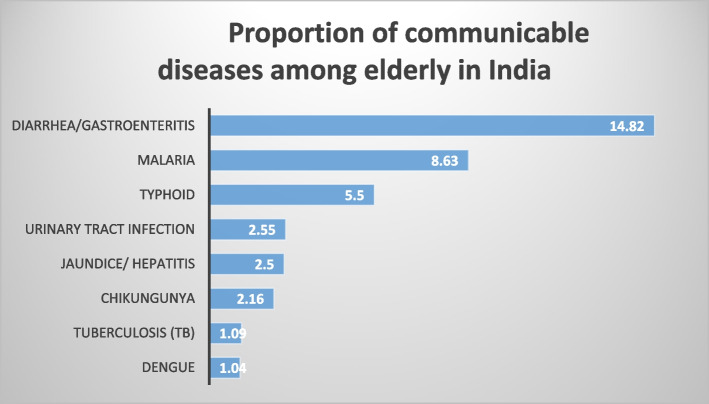
Proportion of communicable diseases among elderly

Figure 1 depicts the prevalence of CDs among the elderly in India. Almost 15 percent of the elderly reported Diarrhoea and another 9 percent reported Malaria. Almost 6 percent of the elderly reported Typhoid.

**Fig. 2:**
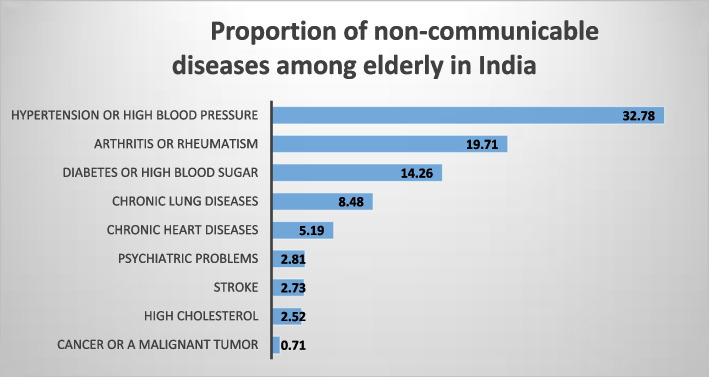
Proportion of non-communicable diseases among elderly

Figure 2 depicts the prevalence of NCDs among the elderly in India. Almost one-third of the elderly reported hypertension (32.8%), and another one-fifth (19.7%) reported Arthritis. In addition, nearly 14 percent reported Diabetes, and 9 percent reported chronic lung diseases. Supplementary tables 1 and 2 depict prevalence of CD and NCD, respectively, by sex of the respondents and ICD-10 codes.

Table 1 depicts the prevalence of communicable and non-communicable diseases among the elderly by various socioeconomic and health characteristics of the elderly. Results found that a higher proportion of female elderly reported communicable (26.8% vs. 26.2%) and non-communicable diseases (55.6% vs. 50.3%) than their male counterparts. Prevalence of communicable diseases was higher among uneducated elderly than those with higher education (29.8% vs. 16.6%); however, the prevalence of non-communicable diseases was higher among those with higher education (67.4% vs. 47.1%) than uneducated elderly. Those who reported good self-rated health had a lower prevalence of communicable (24.9% vs. 36.9%) and non-communicable diseases (50% vs. 70.4%) than those who reported poor self-rated health. Similarly, communicable and non-communicable diseases were higher among those who had severe ADL and IADL disabilities.

**Table 1:** Proportion of communicable diseases (CDs) and non-communicable diseases (NCDs) among elderly by socio-economic and health parameters

	Communicable Diseases (%)	CI (Lower limit)	CI (upper limit)	Non-communicable Diseases (%)	CI (Lower limit)	CI (upper limit)	Total (N)
**Sex**							
Male	26.2	25.0	27.5	50.3	48.7	51.8	14,931
Female	26.8	25.6	28.1	55.6	53.9	57.2	16,533
**Age**							
60–69	26.7	25.6	27.7	50.8	49.5	52.2	18,410
70 +	26.4	24.9	27.8	56.2	54.2	58.2	13,054
**Marital Status**					0.0		
Currently married	26.9	25.9	28.0	51.7	50.4	53.1	19,536
Never Married	30.9	23.1	40.0	45.7	36.8	54.9	225
Divorced/Separated/Diserted	25.9	24.4	27.4	55.4	53.4	57.5	11,703
**Education**							
No education	29.8	28.7	31.0	47.1	45.8	48.4	17,782
Below primary	24.9	22.6	27.4	58.4	55.6	61.2	3,598
Primary	24.2	22.0	26.6	60.2	57.5	62.8	3,520
Secondary	20.5	18.3	22.9	61.2	56.9	65.3	5,285
Higher	16.6	13.8	19.8	67.4	62.8	71.7	1,278
**Living arrangements**							
Living alone	26.5	23.1	30.3	55.3	51.4	59.1	1,787
With spouse	27.0	26.0	28.1	51.6	50.2	53.0	19,176
With others	25.7	24.2	27.3	55.3	53.1	57.5	10,501
**Place of residence**			0.0				
Rural	29.9	29.0	30.9	47.3	46.2	48.3	22,196
Urban	18.4	29.0	30.9	66.9	46.2	48.3	9,268
**Currently working**		0.0					
Yes	28.0	26.6	29.5	29.4	38.2	41.5	9,483
No	26.2	25.0	27.6	28.1	56.4	59.8	13,197
**Wealth Index**							
Poorest	27.4	25.7	29.2	44.8	42.9	46.8	6,829
Poorer	28.8	27.1	30.6	49.9	47.9	51.9	6,831
Middle	24.7	22.8	26.7	51.3	48.4	54.1	6,590
Richer	25.1	23.1	27.1	58.4	55.7	61.2	6,038
Richest	26.5	24.3	28.8	64.0	61.2	66.7	5,175
**Self-rated health**							
Poor	36.9	34.6	39.2	70.4	68.1	72.6	4,630
Good	24.9	24.0	25.8	50.0	48.7	51.2	26,181
**Physical activities**		0.0	0.0			0.0	
Yes	29.2	27.7	30.7	44.4	42.5	46.2	9,704
No	25.6	24.6	26.6	57.4	56.1	58.7	21,494
**Tobacco Use**							
No	24.7	23.6	25.9	56.9	55.4	58.4	18,665
Yes	29.6	28.4	30.9	48.0	46.6	49.4	12,539
**Alcohol use**			0.0			0.0	
Yes	27.4	25.5	29.3	47.3	45.1	49.5	4,555
No	26.6	25.7	27.6	54.4	53.1	55.6	26,655
**ADL disability**							
Severe ADL	32.5	27.9	37.5	71.7	67.0	76.0	999
Moderate ADL	30.3	28.3	32.4	62.5	60.2	64.8	6,045
No ADL	25.6	24.7	26.6	50.3	49.1	51.6	24,291
**IADL disability**							
Severe IADL	31.4	27.8	35.3	61.9	57.9	65.7	1,859
Moderate IADL	29.6	28.1	31.1	58.5	56.6	60.3	13,281
No IADL	23.8	22.8	24.9	48.2	46.9	49.5	16,164
**Total**	**26.7**	**24.7**	**25.6**	**53.1**	53.6	**54.7**	**31,464**

Table 2 depicts the state-wise prevalence of communicable and non-communicable diseases among the elderly. The highest prevalence of CDs was recorded in Dadra & Nagar Haveli (48.3%), followed by Chhattisgarh (47.2%), Rajasthan (46.2%), Haryana (45.8%), Madhya Pradesh (43.5%), Mizoram (41.5%), and Uttar Pradesh (41.1%). Similarly, the highest prevalence of NCDs was recorded in Kerala (78.8%), Goa (72.2%), Andaman & Nicobar Island (68.5%), Lakshadweep (67.9%), Punjab (67.3%), Telangana (66.9%), Puducherry (66.8%), Jammu & Kashmir (66.4%), and Chandigarh (65.7%).

**Table 2:** State-wise prevalence of communicable and non-communicable disease among elderly

**States**	**CDs**	**NCDs**	Total
Jammu & Kashmir	12.9	66.4	321
Himachal Pradesh	31.7	56.2	200
Punjab	27.5	67.3	805
Chandigarh	20.9	65.7	21
Uttarakhand	16.8	50.4	270
Haryana	45.8	58.6	573
Delhi	33.7	62.1	385
Rajasthan	46.2	51.9	1742
Uttar Pradesh	41.1	38.8	4831
Bihar	39.9	43.5	2660
Arunachal Pradesh	33.1	37.4	16
Nagaland	4.8	22.6	45
Manipur	25.9	44.3	69
Mizoram	41.5	49.4	24
Tripura	17	53.4	81
Meghalaya	14.1	39.6	50
Assam	14.2	48.3	525
West Bengal	16.6	65.4	2310
Jharkhand	29	39.1	791
Odisha	20.1	43.4	1201
Chhattisgarh	47.2	33.9	535
Madhya Pradesh	43.5	36.8	1795
Gujarat	30.3	54.1	1408
Daman & Diu	20.8	63.6	4
Dadra & Nagar Haveli	48.3	47.4	5
Maharashtra	15.8	62.5	3327
Andhra Pradesh	11.9	65.2	1423
Karnataka	14.7	53.7	1342
Goa	7.6	72.8	43
Lakshadweep	4.2	67.9	2
Kerala	9.3	78.8	1244
Tamil Nadu	11	63.4	2410
Puducherry	7.3	66.8	42
Andaman & Nicobar Island	28.4	68.5	9
Telangana	14.4	66.9	952

Table 3 depicts the multiple logistic regression of CDs and NCDs among the elderly in India. The odds of NCDs were higher among female elderly (OR = 1.13; C.I. = 1–1.27) than their male counterparts. The odds of CDs decreased with an increase in education; however, the odds of NCDs increased with an increase in education. The results found that the odds of CDs were lower among higher educated elderly (OR = 0.62; C.I. = 0.47–0.81) than uneducated elderly, and odds of NCDs were higher among higher educated elderly (OR = 1.80; C.I. = 1.37–2.35) than their uneducated counterparts. Similarly, the odds of CDs were lower among urban elderly (OR = 0.67; C.I. = 0.59–0.76) than rural elderly, and odds of NCDs were higher among urban elderly (OR = 1.85; C.I. = 1.62–2.10) than their rural counterparts. The results were insignificant for the association between CDs and wealth index; however, the odds of NCDs were higher among the richest elderly (OR = 1.93; C.I. = 1.63–2.28) than the poorest elderly. The odds of CDs (OR = 0.59; C.I. = 0.51–0.68) and NCDs (OR = 0.47; C.I. = 0.41–0.54) were lower among those with good self-rated health than those with poor self-rated health. The odds of NCDs (OR = 1.16; C.I. = 1.03–1.29) were higher among the elderly with no physical activity than their counterparts. The odds of NCDs were lower among the elderly who had no ADL (OR = 0.52; C.I. = 0.35–0.78) than those who had severe ADL limitations.

**Table 3:** Multivariatele logistic regression of communicable diseases (CDs) and non-communicable diseases (NCDs) among elderly by socio-economic and health parameters

	Communicable Diseases		Non-communicable Diseases	
	OR	CI at 95%	OR	CI at 95%
**Sex**				
Male				
Female	0.97	0.88–1.10	1.13^**^	1.00–1.27
**Age**				
60–69				
70 +	0.93	0.83–1.03	1.03	0.93–1.14
**Marital Status**				
Currently married				
Never Married	1.42	0.79–2.54	0.69	0.38–1.21
Divorced/Separated/Deserted	1.11	0.74–1.64	0.91	0.62–1.32
**Education**				
No education				
Below primary	0.85^**^	0.72–0.99	1.52^***^	1.30–1.77
Primary	0.80^**^	0.67–0.94	1.64^***^	1.41–1.92
Secondary	0.77^***^	0.67–0.90	1.75^***^	1.52–2.02
Higher	0.62^***^	0.47–0.81	1.80^***^	1.37–2.35
**Living arrangements**				
Living alone				
With spouse	1.23	0.79–1.91	0.88	0.58–1.32
With others	1.08	0.85–1.36	0.84	0.68–1.02
**Place of residence**				
Rural				
Urban	0.67^***^	0.59–0.76	1.85^***^	1.62–2.10
**Currently working**				
Yes				
No	1.01	0.90–1.13	1.60^***^	1.44–1.79
**Wealth Index**				
Poorest				
Poorer	1.07	0.93–1.23	1.34^***^	1.17–1.53
Middle	0.93	0.79–1.07	1.48^***^	1.28–1.71
Richer	0.92	0.79–1.06	1.68^***^	1.45–1.95
Richest	1.05	0.88–1.23	1.93^***^	1.63–2.28
**Self-rated health**				
Poor				
Good	0.59^***^	0.51–0.68	0.47^***^	0.41–0.54
**Physical activities**				
Yes				
No	0.82^***^	0.73–0.91	1.16^***^	1.03–1.29
**Tobacco Use**				
No				
Yes	1.18^***^	1.06–1.31	0.86^***^	0.78–0.95
**Alcohol use**				
Yes				
No	1.07	0.94–1.21	1.03	0.91–1.15
**ADL disability**				
Severe ADL				
Moderate ADL	1.03	0.69–1.51	0.69	0.46–1.03
No ADL	1.00	0.67–1.46	0.52^***^	0.35–0.78
**IADL disability**				
Severe IADL				
Moderate IADL	1.09	0.81–1.45	1.50^*^	1.12–2.02
No IADL	0.92	0.68–1.24	1.06	0.78–1.43

^***^p < 0.01

^**^p < 0.05

^*^p < 0.10

Table 4 depicts the rural–urban inequality in the prevalence of CDs among the elderly by various characteristics. Results found that education (50%) contributes nearly half of the rural–urban inequality in the prevalence of CDs among the elderly. Self-rated health was another significant predictor that explained nearly one-sixth (16.01%) of the rural–urban inequality in the prevalence of CDs among the elderly in India.

**Table 4:** Decomposition results of rural–urban differentials for communicable diseases (CDs) among elderly by socio-economic and health parameters

	**Coefficient**	**Standard Error**	**Lower limit at 95%**	**Upper limit at 95%**	**Percent contribution**
Sex	0.0011	0.0017	-0.0022	0.0043	-3.72
Age	0.0006	0.0006	-0.0006	0.0018	-2.04
Marital Status	0.0002	0.0008	-0.0014	0.0019	-0.80
Education^**^	-0.0145	0.0068	-0.0278	-0.0013	49.95
Living arrangements	0.0008	0.0012	-0.0016	0.0032	-2.64
Currently working	-0.0037	0.0028	-0.0091	0.0018	12.65
Wealth Index	0.0002	0.0005	-0.0008	0.0013	-0.84
Self-rated health^***^	-0.0047	0.0010	-0.0067	-0.0026	16.01
Physical activities	0.0009	0.0026	-0.0042	0.0061	-3.24
Tobacco Use^**^	-0.0063	0.0027	-0.0116	-0.0009	21.52
Alcohol use	0.0004	0.0006	-0.0007	0.0015	-1.47
ADL disability	-0.0006	0.0006	-0.0018	0.0006	2.10
IADL disability	-0.0035	0.0032	-0.0099	0.0029	12.05

^***^p < 0.01

^**^p < 0.05

^*^p < 0.10

Table 5 depicts the rural–urban inequality in the prevalence of NCDs among the elderly by various characteristics. Education status and current working status were the two significant predictors of widening rural–urban inequality in the prevalence of NCDs among the elderly. On the other hand, wealth index, Self-rated health, and IADL disability were the three factors narrowing down the rural–urban inequality in the prevalence of NCDs among the elderly in India.

**Table 5:** Decomposition results of rural–urban differentials for non-communicable diseases among elderly by socio-economic and health parameters

NCDs	Coefficient	Standard error	Lower limit at 95% CI	Upper limit at 95% CI	Percent contribution
Sex	-0.0020	0.0022	-0.0064	0.0023	-5.6
Age	-0.0010	0.0007	-0.0024	0.0003	-2.8
Marital Status	0.0010	0.0007	-0.0004	0.0025	2.9
Education^***^	0.0370	0.0099	0.0176	0.0565	102.6
Living arrangements	-0.0008	0.0010	-0.0028	0.0011	-2.3
Currently working^***^	0.0108	0.0038	0.0034	0.0183	30.0
Wealth Index^**^	-0.0018	0.0009	-0.0035	-0.0001	-5.0
Self-rated health^***^	-0.0053	0.0011	-0.0075	-0.0031	-14.6
Physical activities	0.0035	0.0038	-0.0039	0.0108	9.6
Tobacco Use	0.0039	0.0037	-0.0033	0.0110	10.7
Alcohol use	0.0004	0.0006	-0.0009	0.0016	1.0
ADL disability	-0.0016	0.0010	-0.0035	0.0003	-4.4
IADL disability^**^	-0.0081	0.0041	-0.0160	-0.0001	-22.3

^***^p < 0.01

^**^p < 0.05

^*^p < 0.10

## Discussion

Our study attempts to assess the prevalence of CDs and NCDs among the elderly and its associated factors. In this study, we found that CDs and NCDs are burdening a substantial proportion of the elderly in India. The NCDs have been acknowledged as an emerging global health concern since the beginning of the twenty-first century [21], and also it has already started sweeping extensively all over the world with an exceedingly increasing trend in developing countries [11, 21, 22]. However, India does not recognize it as an important public health challenge till the second decade of this millennium [23]. Despite NCDs' lack of significance in the National Health Policy 2002, they received special attention in the Draft National Health Policy 2015 [23].

This paper envisages the prevalence of both types of diseases among the elder population and found that more than half of the elderly population suffers from NCD, and nearly one-third suffers from CD. A study based on wave one of the world Health organization's study on global ageing and adult health also found the same pattern of NCD’s prevalence and reported that 50% of total aged population is suffering from at least one type of chronic non-communicable disease [24]. Furthermore, in a report released by Ministry of Health and Family Welfare (MOHFW), Government of India (GOI), “India: Health of the Nation’s States” reveal that NCDs' contribution to total disease burden-'disability-adjusted life years' (DALYs) increased from 30 percent in 1990 to 55 percent in 2016, as well as the proportion of fatalities owing to NCDs (in total deaths) increased from 37 percent in 1990 to 61 percent in 2016. This demonstrates a rapid epidemiological shift in illness burden to NCDs.

Also, the prevalence of these diseases varies with the socio-economic and bio-demographical backgrounds of the elderly. The percentage of elderly suffering from NCD is higher among those who are highly educated, richest, living in the urban area, taking alcohol, having severe ADL and IADL, whereas CD is common among the elderly those who are uneducated and lower educated, living in rural areas, poorer, taking tobacco, having severe ADL and IADL. Additionally, it has been noted that older people living alone have greater rates of CD and NCD. The living arrangements of the older population have a significant impact on their health. The percentage of elders with CDs and NCDs is higher among those who are living alone. The fertility decline has a direct association with the declining co-residence of the elders [25], and as a consequence, the elderly are not getting the care that is required for their better health status.

People with higher levels of education have better knowledge acquisition abilities, which makes them more likely to identify disease symptoms, report them, and seek treatment from medical institutions more immediately [26]. Therefore, it is considered that a person's higher level of education serves as a safety net for them and lowers their risk of contracting communicable diseases. Since educated people are more likely to be wealthy and have sedentary lifestyles, which increase their risk of non-communicable illness, they have higher probabilities of developing NCD [27–29].

Similarly, the odds of CD were lesser among older adults living in urban areas whereas odds of NCD were higher among urban residents as compared to their counterparts. Sanitation is an important aspect in the occurrence of communicable [30], and it is evident in rural areas sanitation facilities are poorer than in urban areas [31] which can be linked to higher CD among older adults in rural areas than in urban areas. Older adults in urban areas follow a sedentary lifestyle which is why they have higher odds of NCD than their rural counterparts [27, 28]. Results noticed slightly lower odds for NCDs among those who consume tobacco than their counterparts. This finding deviates from previous studies [32, 33] and the possible causes are unexplored for the same.

The study also presents state-wise variation in CDs and NCDs among the elderly. The NCDs are reported to be more prevalent in south Indian states, and among all Indian states, UTs and Kerala have the greatest percentage of older people who have NCDs. The high burden of NCD in Kerala among the elderly is primarily due to the increase in the proportion of their population and the adoption of a sedentary lifestyle [34]. The burden of CDs are found to be higher in Chhattisgarh, Rajasthan, Haryana, Madhya Pradesh, Uttar Pradesh, and Bihar. The geographical pattern of CDs and NCDs manifests the north–south divide in the burden of these diseases. The less-developed north India with a lower percentage of urbanization has more burden of CDs, whereas the more developed southern India has a higher percentage of the urban population, has a greater burden of NCDs. As reported by the previous literature, the urban population has a greater burden of NCD and related risk [35]. Furthermore, regional differences in disease prevalence may be explained by the region's eating habits and dietary practices, tobacco consumption, and sedentary habits [36].

The present study also shows the proportion of CDs and NCDs among the elderly in India. Diarrhoea, malaria, and typhoid make up the majority of CDs, while Chikungunya, tuberculosis, and dengue make up the minority. The most common NCDs are hypertension, arthritis, diabetes, and chronic lung diseases. The hearth diseases and cancer have a comparatively lower prevalence than above-mentioned NCDs, but they are found to be most fatal across countries. Globally, the heart diseases (cardiovascular diseases) has the highest fatality rate among all NCDs and account for nearly 17.9 million death annually which is followed by death because of cancers (9.3 million), chronic lung diseases (4.1 million), and diabetes (1.5 million) [37]. In India, these four NCDs, including stroke, account for nearly 5.8 million deaths annually [21, 38]. On the other hand, with these NCDs, the CDs continue to pose a significant challenge to India’s elderly life.

Education was identified as one of the key contributors to the CD and NCD disparity between urban and rural areas among older persons. In India, the importance of education is widely studied in relation to healthcare among older adults [27, 28, 39, 40]. A person's education influences their awareness and helps them accept a diagnosis and make the necessary behavioural changes [41]. However, in one of the South African studies, education was not a significant predictor of non-communicable diseases among older adults [42].

Corroborating with previous findings [43, 44], the study noted a higher likelihood of NCDs among the female elderly than their male counterparts. In developing countries, including India, women report more about symptoms of their illness than men, which could be attributed to their higher prevalence of disease [27, 45]. Also, it has been noted that females tend to suffer from chronic debilitating conditions but not fatal ones, and this explains the paradox of high morbidity and less mortality among them compared to men [46]. In line with previous studies [28, 44], the study noted a higher odds of CDs among rural elderly, whereas the risk of NCDs was higher among urban elderly than their respective counterparts. A sedentary lifestyle and physical inactivity could expose the urban population to a high risk of NCDs [47, 48]. Furthermore, nuclear family setup causing loneliness lack of care could be another reason of high NCDs among the urban population [49]. The findings of higher odds of NCDs among highly educated and richest elderly agree with previous literature [28]. Elderly people who are educated and wealthy are more likely to lead sedentary lifestyles, which may be a contributing factor to greater NCD rates.

## Strengths and limitations

The study has some potential limitations. The study has attempted to fill in the literature gap by examining the CDs and NCDs in a single study among the elderly in India using a nationally representative sample survey-based data. Despite its considerable strength, the study has few significant limitations. The cross-sectional nature of data limits our understanding of causal inferences. Moreover, the reporting of NCDs could be affected by the recall bias. Self-rated health as a predictor variable was an outcome of self-reporting of health by the older adults and therefore it can be affected by recent events pertaining to healthcare, thereby providing some false implications. Also, this study captured information about the consumption of alcohol and tobacco by the older adults but failed to quantify the information. It is evident that quantity in terms of alcohol and tobacco consumption plays an important role in deciding the health outcomes including NCD to a greater extent. Further, the association between ADL and IADL with communicable and non-communicable diseases is not conclusive as disability could be secondary to the NCD or as a consequence of the NCD and not necessarily contributory. There is much debate on whether to include Jaundice/Hepatitis as communicable disease or not and we have categorized it as communicable disease.

## Conclusion

The burden of both CD and NCD among the elderly population requires immediate intervention. Among both types of diseases, the NCD is recognized as a more fatal and long-duration disease resulting from a combination and role of physiological, environmental, behavioural, and genetic factors throughout the life cycle. Although NCDs are treatable once diagnosed but as a prolonged health condition due to the sedentary lifestyle, it cannot be halted by providing treatment. Progression to complications or end-stage organ damage in the case of diabetes and hypertension could well be prevented by proper control. NCD is more of a concern in urban areas than in rural areas. Sedentary lifestyle behaviour. Urbanization has been associated with NCD risk factors such as low physical activity, unhealthy diet, overweight and high blood pressure. It is imperative that investment shall be made to promote physical activity and healthy lifestyle. Similarly, CDs were more of a concern in rural areas than in urban areas and interventions are to be promoted in rural areas to tackle CDs. The needs of men and women and urban and rural elderly must be addressed through appropriate effort. In a developing country like India, preventive measures, rather than curative measures of communicable diseases, will be cost-effective and helpful.

## Supplementary Information

**Additional file 1:** 

**Additional file 2:** 

## Data Availability

The datasets generated and/or analysed during the current study are available with the International Institute for Population Sciences, Mumbai, India repository and could be accessed from the following link: https://iipsindia.ac.in/sites/default/files/LASI_DataRequestForm_0.pdf. Those who wish to download the data have to follow the above link. This link leads to a data request form designed by International Institute for Population Sciences. After completing the form, it should be mailed to: datacenter@iips.net for further processing. After successfully sending the mail, individual will receive the data in a reasonable time.

## Abbreviations

ADL: Activity of Daily Living
CD: Communicable Disease
CEB: Census Enumeration Block
CI: Confidence Interval
DALYs: Disability-adjusted Life Years
GoI: Government of India
HRS: Health and Retirement Study
HSPH: Harvard T.H. Chan School of Public Health
IADL: Instrumental Activity of Daily Living
IIPS: International Institute for Population Sciences
LASI: Longitudinal Ageing Study in India
MoHFW: Ministry of Health and Family Welfare
NCD: Non-communicable disease
NIA: National Institute on Aging
OR: Odds Ratio
TB: Tuberculosis
UNFPA: United Nations Population Fund
USC: University of Southern California
UTs: Union Territories
