# Supplementary material for: Burden of communicable and non-communicable diseases-related inequalities among older adults in India: a study based on LASI survey
Source: BMC Geriatr. 2022 Oct 10;22:790. doi: 10.1186/s12877-022-03481-x (PMC9552506; doi:10.1186/s12877-022-03481-x)
Supplement: Supplementary file 1 — Additional file 1: [file 12877_2022_3481_MOESM1_ESM.docx]

| Supplementary Table 1: Prevalence of communicable diseases (CDs) by sex of the respondents and its ICD-10 codes | | |
| --- | --- | --- |
|  | Male | Female |
| Jaundice/Hepatitis (R17, B18.2) | 2.6 | 2.3 |
| Tuberculosis (A16-A19) | 1.3 | 0.9 |
| Malaria (B54) | 8.4 | 8.8 |
| Diarrheal/gastroenteritis (R19.7, K52.9) | 14.7 | 14.8 |
| Typhoid (A01.0) | 4.8 | 6.1 |
| Urinary tract infection (N39. 0) | 3.0 | 2.0 |
| Chikanguniya (A92. 0) | 2.1 | 2.2 |
| Dengue (A90) | 0.7 | 1.2 |
| **Total** | **14,930** | **16,533** |
