# Supplementary material for: Burden of communicable and non-communicable diseases-related inequalities among older adults in India: a study based on LASI survey
Source: BMC Geriatr. 2022 Oct 10;22:790. doi: 10.1186/s12877-022-03481-x (PMC9552506; doi:10.1186/s12877-022-03481-x)
Supplement: Supplementary file 2 — Additional file 2: [file 12877_2022_3481_MOESM2_ESM.docx]

| Supplementary Table 2: Prevalence of non-communicable diseases (NCDs) by sex of the respondents and its ICD-10 codes | | |
| --- | --- | --- |
|  | Male | Female |
| Hypertension (I10, I12, I15) | 27.9 | 37.1 |
| Diabetes (E10 - E14) | 14.6 | 13.9 |
| Cancer or a malignant tumour (C00-C97) | 0.6 | 0.8 |
| Chronic lung disease | 9.0 | 8.0 |
| Chronic heart disease (I00 - I09, I11, I13, I20 - I51) | 5.8 | 4.6 |
| Stroke (I60 - I69) | 3.2 | 2.2 |
| Arthritis or rheumatism (M06) | 16.2 | 22.7 |
| Psychiatric problems (F00-F99) | 2.7 | 2.9 |
| High cholesterol (E78) | 2.6 | 2.3 |
| **Total** | **14,930** | **16,533** |
